# Abnormal topological organization of the white matter network in Mandarin speakers with congenital amusia

**DOI:** 10.1038/srep26505

**Published:** 2016-05-23

**Authors:** Yanxin Zhao, Xizhuo Chen, Suyu Zhong, Zaixu Cui, Gaolang Gong, Qi Dong, Yun Nan

**Affiliations:** 1 State Key Laboratory of Cognitive Neuroscience and Learning & IDG/McGovern Institute for Brain Research, Beijing Normal University, Beijing, China.

## Abstract

Congenital amusia is a neurogenetic disorder that mainly affects the processing of musical pitch. Brain imaging evidence indicates that it is associated with abnormal structural and functional connections in the fronto-temporal region. However, a holistic understanding of the anatomical topology underlying amusia is still lacking. Here, we used probabilistic diffusion tensor imaging tractography and graph theory to examine whole brain white matter structural connectivity in 31 Mandarin-speaking amusics and 24 age- and IQ-matched controls. Amusics showed significantly reduced global connectivity, as indicated by the abnormally decreased clustering coefficient (C_p_) and increased normalized shortest path length (λ) compared to the controls. Moreover, amusics exhibited enhanced nodal strength in the right inferior parietal lobule relative to controls. The co-existence of the lexical tone deficits was associated with even more deteriorated global network efficiency in amusics, as suggested by the significant correlation between the increments in normalized shortest path length (λ) and the insensitivity in lexical tone perception. Our study is the first to reveal reduced global connectivity efficiency in amusics as well as an increase in the global connectivity cost due to the co-existed lexical tone deficits. Taken together these results provide a holistic perspective on the anatomical substrates underlying congenital amusia.

Music constitutes an essential part of our lives. However, approximately 4% of the general population experiences a lifelong disorder in music perception and production, which is a neurodevelopmental condition known as congenital amusia (amusia or amusics hereafter)^1^. These individuals demonstrate a characteristic musical pitch deficit^2^, with no clear neurological origin nor due to a lack of musical experiences. The pitch deficits in amusics might be related to some regional structural abnormalities (e.g., altered white and grey matter in the bilateral superior temporal and inferior frontal regions of amusics’ brains)^3^,^4^,^5^. Interestingly, recent studies have shown that the associated abnormalities may reside not only in isolated brain regions but also in some connective pathways (e.g., the temporo-frontal pathway that connects the auditory cortex to the inferior frontal region)^6^,^7^,^8^,^9^ or regional brain network (e.g., dorsolateral prefrontal cortex network)^10^.

The above imaging results have indicated that amusia might be a disorder of brain connectivity, affecting neural networks that are crucial for pitch-related processing rather than some isolated brain area. However, to the best of our knowledge, all of the previously published imaging studies concerning amusics were based on populations speaking non-tone languages. How amusia might affect the brain network topologies of tone language speakers remains unknown. Importantly, recent studies have found that similar cases of amusia also exist under the tonal language environment^11^,^12^,^13^,^14^. More importantly, despite tone language experiences, these amusics may also show behavioural difficulties in processing lexical tones, either at the whole group level^12^ or within a subgroup^11^,^13^,^14^.

The current study aimed to investigate the brain network properties of Mandarin-speaking amusics. Understanding the brain network traits in Mandarin amusics that underlie not only musical pitch deficits but also lexical tone difficulties will expand our knowledge on the nature of amusia and ultimately inform us on the neural mechanisms of musical pitch and lexical tone processing as well as the debatable relationship between music and speech.

An optimal approach to quantify the anatomical connectivity pattern that is unique in amusics is to use graph theoretical analysis to comprehend the related properties in complex networks^15^. Previous studies have demonstrated that a brain structural network can be constructed using noninvasive diffusion tensor imaging (DTI) at the macroscale^16^ and can be further analysed based on graph theory to reveal the connectivity patterns of complex networks^15^.

In the current study, we used diffusion probabilistic tractography and graph theoretical analysis to examine the topological characteristic of white matter networks in Mandarin-speaking amusics relative to matched controls. In this regard, we focused on the differences of small-world properties between two groups at the global level. Briefly, the small-world structure of a brain network can be reflected by normalized shortest path length λ and normalized clustering coefficient γ, which indicate the global efficiency and average local efficiency for the transformation of information. We also compared the non-normalized form of the above two properties (namely L_p_ and C_p_), considering the possible effects of normalization on the significance of our results. At the regional level, we mainly focused on the nodal strength (or nodal degree in binary network) which is the most direct index to reflect the connection between a certain node and all the other nodes. Although the voxel-based studies of amusics reported structural abnormalities in focal brain regions such as superior temporal and inferior frontal areas^3^,^4^,^5^, functional and structural connectivity studies have demonstrated that the key neural mechanisms underlying amusia might be the reduced connectivity between these regions^6^,^7^,^8^. The structural abnormality may reside in the right arcuate fasciculus (AF), a white matter tract that connects the right superior temporal and inferior frontal regions^6^. Although a recent study did not replicate the structural disorder in the AF for the amusics^17^, an intervention study showed that the application of low gamma oscillations to the right dorsolateral prefrontal cortex with transcranial alternating current stimulation (tACs) would improve pitch memory performance in amusics possibly by modulating the connectivity of the right AF^10^. Long-range connections such as the right AF are very important in keeping the global connectivity efficient^15^, although they are few in number. Given that amusics have shown defected right AF both functionally and structurally^6^,^7^,^8^, we hypothesized that the global connectivity efficiency would be lower in amusics relative to controls. Due to the impaired right AF, information flow within the right fronto-temporal areas might rely more on the indirect routes – two short pathways that unite the frontal and temporal regions via the inferior parietal lobe^15^,^18^. As a result, we expected increased local connectivity between this area and the neighbouring areas in amusic than the controls. In addition, the co-existed lexical tone deficits in some of the amusics^11^,^13^,^14^ would also disrupt the topological organization of the white matter network. Since the processing of lexical tones implicates bilateral brain network^19^,^20^, we expected that the lexical tone deficits might deteriorate the global connectivity deficiency in amusics.

## Results

### Demographic and behavioural characteristics

There were no significant group differences in gender, age, IQ, and brain size between the controls and amusics. The amusics had significantly lower scores on the MBEA test and lexical tone test (both *p*s < 0.001) compared to controls (Table 1).

### Comparison of network properties

Both the amusics and controls showed typical small-world organization for their white matter weighted brain networks as reflected by the normalized clustering coefficient γ ≫ 1 (Multi-threshold Mean ± SD: amusics: 4.054 ± 0.113, controls: 4.027 ± 0.104) and the normalized shortest path length λ ≈ 1 (Multi-threshold Mean ± SD, amusics: 1.280 ± 0.015, controls: 1.271 ± 0.015).

**Figure 1:**
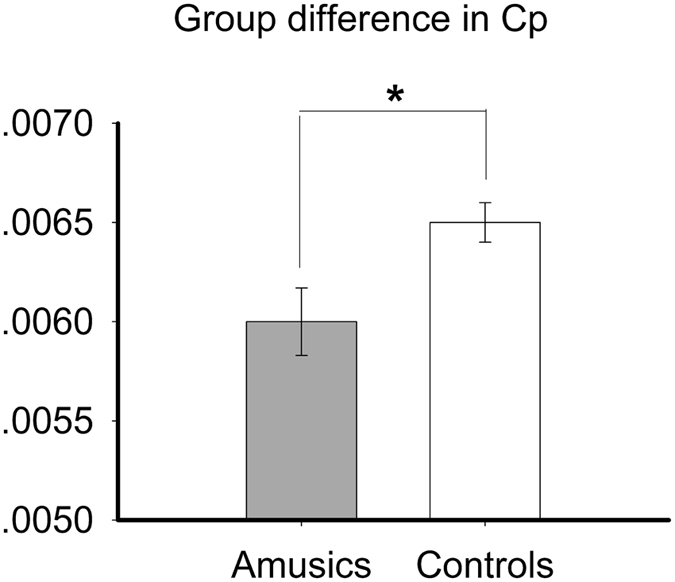
Clustering coefficient (Cp) of the area under curve (AUC) of the amusics and the controls. Error bar represents standard error. *p < 0.05.

**Figure 2:**
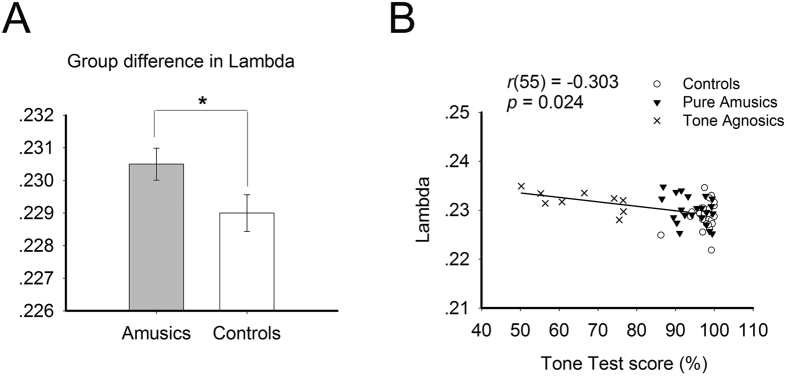
(**A**) Lambda of the area under curve (AUC) of the amusics and the controls. Error bar represents standard error. *p < 0.05. (**B**) Correlation between Lambda and the Tone Test score for all participants.

We found a significant increase in the normalized shortest path length λ (*p* = 0.031) and a significant decrease in the clustering coefficient C_p_ (*p* = 0.012) for amusics relative to controls (Figs 1 and 2A). No group difference was found for the shortest path length L_p_ and normalized clustering coefficient γ, both *p*s > 0.1.

The observed group differences in the clustering coefficient (C_p_) but not in the normalized clustering coefficient (γ) and in the normalized shortest path length (λ) but not in the shortest path length (L_p_) are reminiscent of a previous study^21^ where group differences were observed with the shortest path length (L_p_) but not with the normalized shortest path length (λ). Though C_p_ and γ, as well as L_p_ and λ have similar physiological meaning, technically, C_p_ and L_p_ are more parallel with absolute values (average of all clustering coefficients or shortest path lengths), while γ and λ are more parallel with relative values (where C_p_ or L_p_ was divided by the mean value of n random networks). This suggests the necessity to include both the original and the normalized global network parameters in order to fully describe the network properties of the special population such as amusics.

**Figure 3:**
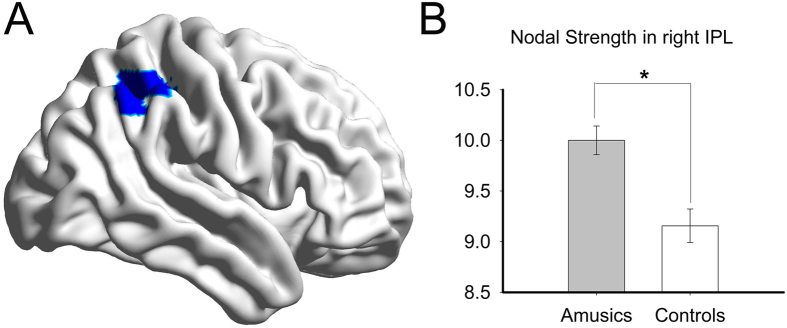
(**A**) The right inferior parietal lobule in which the amusics showed significantly greater nodal strength than the controls (highlighted in blue). (**B**) Nodal Strength of the area under curve (AUC) of the amusics and the controls. Error bar represents standard error. *p < 0.05. Figures 3A and4 were plotted using an in-house software BrainNet Viewer^64^. http://www.nitrc.org/projects/bnv.

Between-group difference of the nodal strength was observed in the right inferior parietal lobule (IPL) (Fig. 3). Amusics exhibited significantly higher nodal strength in the right IPL region compared to controls (*p* = 0.0004). No significant results were found in other nodes or other nodal properties. With regards to the group differences under multiple thresholds (sparsities) of λ, C_p_, and nodal strength of IPL, see Supplementary materials for detail. As shown in Supplementary materials, apparently, the group differences on these measures remain the same regardless of the specific tested threshold level.

### Relationship between behavioural lexical tone perception and network measures

The lexical tone test score was negatively correlated with the normalized shortest path length λ (*r* (55) = −0.303, *p* = 0.024) and the nodal strength of the right IPL region (*r* (55) = −0.307, *p* = 0.023) among all participants. Except for the significantly negative correlation between the lexical tone test score and normalized shortest path length λ (*r* (31) = −0.434, *p* = 0.015) within the amusic group, none of the above observed correlations held in the amusic or control groups. No other significant correlations were observed.

We demonstrated that the MBEA and lexical tone scores were positively correlated with each other in our previous study^11^. In the current study, these two were also highly correlated across all participants (*r* (55) = 0.612, *p* < 0.001). Thus, it was necessary to disentangle the observed behavioural-brain effects for the normalized shortest path length λ and nodal strength of the right IPL region, as these two network measures might be correlated with the lexical tone test scores simply because the lexical tone test scores and the MBEA scores were positively correlated. Partial correlations were performed to examine these complex brain-behaviour associations. When partialling out the effect of lexical tone test score, a significant correlation was found between MBEA score and the nodal strength of the right IPL (*r* (55) = −0.391, *p* = 0.003), while no significant result was found between MBEA score and λ (*r* (55) = −0.068, *p* = 0.626). When partialling out the effect of MBEA score, a significant correlation was found between lexical tone test score and λ (*r* (55) = −0.306, *p* = 0.025), while no significant result was found between lexical tone test score and the nodal strength of the right IPL (*r* (55) = 0.027, *p* = 0.846).

### Hub nodes

**Figure 4:**
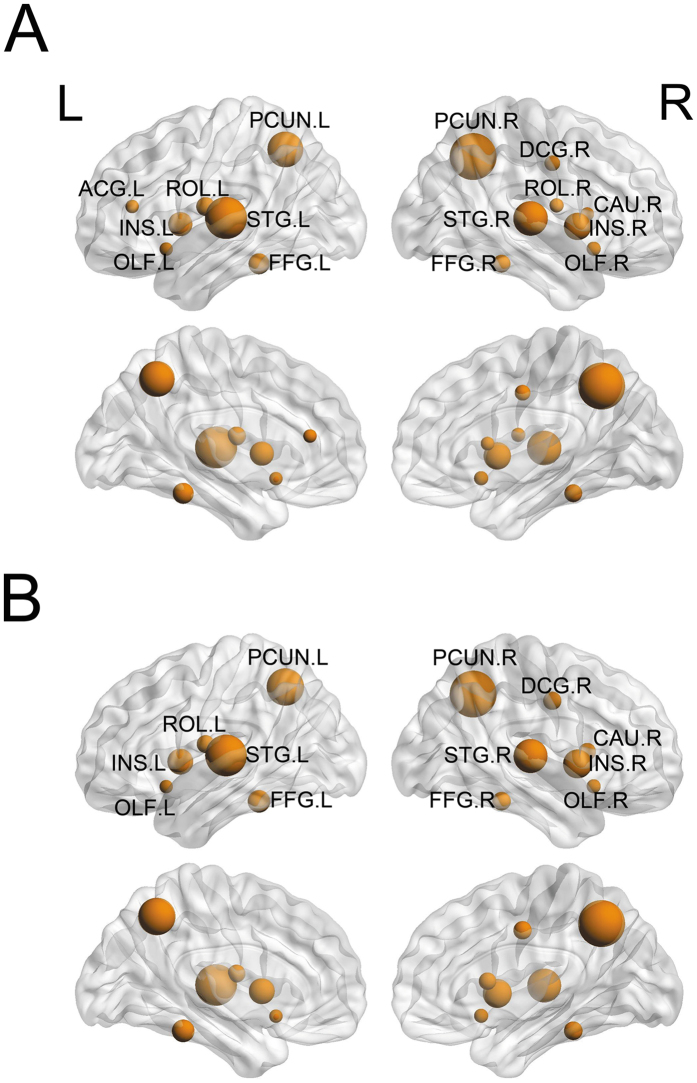
The network hubs with high nodal strength (at least 1 SD higher than the mean AUC of nodal strength) in the amusics (**A**) and the controls (**B**), with nodal size indicating the corresponding normalized AUC of the nodal strength. PCUN, Precuneus; STG, Superior temporal gyrus; INS, Insula; FFG, Fusiform gyrus; ROL, Rolandic operculum; DCG, Median cingulate and paracingulate gyri; OLF, Olfactory cortex; CAU, Caudate nucleus; ACG, Anterior cingulate and paracingulate gyri; R, right; L, left.

We identified the hub nodes using the AUC of the nodal strength of the white matter weighted networks for each group. To define hub nodes, we followed the leniency criterion according to previous studies e.g.^22^: one SD above the mean nodal strength of each group was regarded as the dividing line of hubs and non-hubs. We found that the hubs for the two groups (amusics and controls) were relatively similar. Nodes that had strengths 1 SD greater than the mean included the bilateral precuneus, the superior temporal gyrus, bilateral insula, fusiform gyrus, olfactory cortex, right median cingulate, paracingulate gyri, and left rolandic operculum for both groups (Fig. 4). Our results were partly congruent with previous white matter network studies identifying hubs by high nodal efficiency^22^,^23^,^24^ or using multiple criteria^25^, suggesting that only a minority of nodes in the white matter network has a higher importance in informational integration.

### Effects of weight and resolution

To inspect the effects of weight and resolution upon the observed significant results, we also used binary and high-resolution (AAL-1024) networks.

There was no significant group difference under the binary network condition at both the global and regional levels. This finding suggests that the significant between-group differences obtained in the weighted network was not dependent on the connection number, but was significantly affected by the connection weight, which was indispensable for the cerebral connectivity within the real functioning brain.

Using a high-resolution network, within the region of interest (the right IPL) observed under a resolution of 90, we also found some of the nodes in amusics showing significantly increased nodal strength (*p* < 0.05, uncorrected). However, no significant group difference was found at the global level. Similar differences due to a change in resolution were also found in previous studies using either a white matter network^22^ or a functional network^26^. This was most likely due to low network sparsity (ranging from 5.36% to 7.72% for all raw matrices) caused by the probabilistic tracking algorithm under high resolution, where the proportion of the non-zero connection was too small. Because the white matter fibres deep in our brain have the organizational form of bundles, we believe that a relatively larger nodal size (as those obtained at a 90 resolution) may better resemble the white matter topological properties compared to those at a 1024 resolution.

## Discussion

Using probabilistic DTI tractography and graph theory analysis, the present study revealed alternations in the topological organization of the white matter networks in Mandarin-speaking amusics. Similar to controls, amusics demonstrated an overall intact topology of the small-world structure. This is reminiscent of similar small-world organizations observed in cortical thickness networks across musicians and non-musicians^27^, supporting the notion that the human brain is organized anatomically in accordance to the small-world principle. Moreover, amusics and controls demonstrated quite similar patterns of hub distribution. Some hub nodes found in the present study (e.g. bilateral precuneus and insula) were also reported as hubs in other research of white matter network^22^,^24^. In fact, similar hub patterns were also found between normal people and patients with attention deficit hyperactivity disorder (ADHD), Alzheimer’s disease, remitted geriatric depression, or amnestic mild cognitive impairment^21^,^22^,^24^, suggesting that the hub node is a quite robust property in the organization of the white matter network. However, a closer look suggests that there were still slight differences in network hubs between the amusics and the controls. Some hubs (e.g. the right Rolandic operculum and the left anterior cingulate and paracingulate gyri) in amusics were not hubs for the controls. Previous studies have found that these above two regions were involved in musical emotion processing^28^,^29^. We believe these results might reflect a regional compensative mechanism due to the possible malfunction in some pathways for musical processing in amusics. Moreover, amusics showed greatly reduced global connectivity as indicated by the increased normalized shortest path length and the abnormally decreased clustering coefficient compared to the controls. Furthermore, amusics exhibited enhanced nodal strength in the right inferior parietal lobule relative to controls. The co-existence of the lexical tone deficits was associated with even more deteriorated global network efficiency in amusics, as suggested by the significant correlation between the increments in normalized shortest path length and the insensitivity in lexical tone perception.

Our study is the first study to reveal that the amusics demonstrated reduced global connectivity in white matter structure compared to the controls. This global hypoconnectivity in amusics is reflected by two abnormal small world measures: the significantly increased normalized shortest path length and the significantly decreased clustering coefficient. As expected, the greatly increased normalized shortest path length in amusics relative to the controls is indicative of reduced global connectivity efficiency^15^. This is consistent with the previously observed reduced structural and functional connectivity in the right arcuate fasciculus (AF) for the amusics^6^,^7^,^8^.

The clustering coefficient is a measure of the average local information transferring efficiency of the whole network^15^ and reflects the average connectivity efficiency of all of the local networks in the whole brain. The significantly decreased clustering coefficient in amusics suggests an anomaly of average brain network integrity in amusics as compared to controls. It is conceivable that the structural aberrations in some local brain areas might have contributed to the observed significantly lower clustering in amusics compared to controls. Thus, these data have the same trend with the structural abnormalities previously found in the bilateral inferior frontal gyri (IFG), superior temporal gyri (STG)^3^,^4^,^5^,^8^, and temporo-frontal pathway that connects the auditory cortex to the inferior frontal region in amusics^6^,^7^,^8^.

Overall, a previous study in white matter network has found that the global efficiency was significantly increased during late childhood, while the local efficiency was rapidly increased in adolescence^30^. Thus, the developmental disorder of white matter caused by congenital amusia possibly underlies the observed decrease of white matter network efficiency in the amusics. Chinese dyslexics were also found to have an evident tendency of decrease in structural network efficiency^31^, which might be mainly caused by developmental abnormality of several white matter connections (for a review)^32^. The decrease of white matter network efficiency, as reflected by comparatively lower clustering coefficient and longer path length, was also found in other developmental disorders such as schizophrenia^33^.

The observed increased nodal strength in the right IPL in amusics relative to the controls might suggest that adaptive changes in the brain compensated for the disrupted global connectivity. The fact that this is only observable in the right hemisphere is consistent with previously observed structural anomalies^3^,^4^,^8^ and decreased functional connectivities^7^,^8^ in the right fronto-temporal area in the amusics relative to controls. However, no group differences were found in IFG and STG where structural abnormalities were detected in previous studies at the nodal level. This reflects the fact that the traditional methods focused on impairments within local regions in amusics while the nodal strength in present study reflect an abnormal change of connection between one node and the others.

The inferior parietal lobe plays an important role in music processing^34^. As shown in Fig. 3A, the area with increased nodal strength in the amusic group relative to the control group resides mainly in the supramarginal gyrus of the inferior parietal region. The supramarginal gyrus has been implicated in perception tasks such as musical discrimination for both adults^35^ and children^36^, pitch memory^37^, and rhythm processing^38^. In addition to perception, the supramarginal gyrus also underlies music production^39^. Furthermore, the inferior parietal lobe in general are specifically involved in music memory^40^,^41^,^42^. The impairment of some of these functions is generally linked to amusia. The increase in nodal strength in this area might thus reflect a structural alteration of compensatory nature due to long-term functional adaptation to the compromised pitch-related processes in amusics.

In addition, the increased nodal strength in the right IPL is also consistent with the defected right arcuate fasciculus found in amusics^6^,^43^. The inferior parietal lobule is the pivotal hub that connects two short pathways that unite the frontal and temporal regions^18^, an alternative to the arcuate fasciculus, which is the direct highway bridging these two brain regions. The anterior pathway connects the frontal area and inferior parietal lobe, and the posterior pathway links the inferior parietal lobe with the temporal area^44^. This indirect connection might become an important alternative neural route that is responsible for information communication between the frontal and temporal areas as a substitute for the defective arcuate fasciculus in the brains of amusics. Of note, a previous study^45^ has shown that better pitch-related grammar learner had a higher white matter integrity in the right supramarginal gyrus. Given that our current study found increased nodal strength in the right IPL in the amusics, both of these results highlight the importance of the right IPL as a critical region for pitch processing. However, please note that increased nodal strength in the right IPL for the amusics does not necessarily contradict with higher FA values underlying the right supramarginal gyrus (along the course of ventral arcuate fasciculus, which connected the IFG and MTG) for the good pitch grammar learners. Higher FA values suggest more integrate ventral AF, whereas increased nodal strength in the right IPL indicates more neural pathways connecting to (different from passing through) the right IPL. It is possible that these vital “ventral AF” connections to the right IPL might be malfunctioned in the amusics, hence the nodal strength in this area is increased to compensate. Indeed, as shown in prior work^6^, using tractography initiated in the right STG, the authors found resulted fibers projecting toward the ipsilateral IFG in controls, but for the amusics, the resulted fibers projected dorsally toward the parietal lobe. This is in line with our currently observed increased nodal strength in the right IPL.

In addition to musical pitch deficits, Mandarin-speaking amusics also suffer from lexical tone difficulties, although only in a small subgroup^11^,^13^,^14^. Our results suggest that this behavioural phenotype of lexical tone deficits among Mandarin-speaking amusics is associated with an abnormally increased normalized shortest path length, reflecting the brain’s decreased global connectivity efficiency^15^. The increase in λ is tightly linked to the severity of the lexical tone deficits in amusics: the more severe the tone deficit, the more disrupted the brain’s global connectivity.

Importantly, the lexical tone deficits observed in amusics thus far are limited in perception, but lexical tone production is spared^11^,^13^. The perception of speech sound, such as lexical tones, relies on the functional integration of auditory processing (the temporal area) and articulation (the frontal regions)^46^. Here, the arcuate fasciculus as the neural bundle connecting these areas is indispensable^47^,^48^. The lexical tone deficits may more likely implicate the long-range connections, such as the arcuate fasciculus, causing an increased normalized short path length, which is reflective of a less economical cortical network. Indeed, a similar tendency of increased λ and decreased global efficiency has also been found in children with reading problems in a network study based on grey matter volumetric covariates^31^. This could be associated with defects of long-range connections^49^,^50^, particularly in the left arcuate fasciculus^49^,^51^. Future studies may continue to explore how lexical tone deficits, in contrast to musical pitch deficits, modulate the structural integrity of the arcuate fasciculus.

An important caveat of the current study is about the methodology of probabilistic tractography. Although the problem of fibre crossing has been solved by using probabilistic tractography^52^, it still might introduce spurious connections that do not exist in a real white matter network. Thus, we used a wide range of thresholds to calculate the AUC to minimize the effect.

Importantly, by the general assumption, the group of amusics across cultures is normally free of any neurological deficits, and thus, the expected topological alterations, if any, should not be very large. Indeed, the effect sizes of the observed group differences in brain network patterns in the current study, as indicated by the correlation coefficient, were mostly medium (all *r*s > 0.3) according to Cohen^53^. Our results showed that for amusics with or without lexical tone deficits, there were always some degrees of global network deficiency. This finding supports the notion that the condition of amusia involves spatially distributed brain networks rather than some focal brain areas. Converging with previous studies^6^,^43^, our results point to the most important long-range neural tract – the arcuate fasciculus – as one of the candidates for future investigations in neural mechanisms of amusia. It would be interesting to understand how musical pitch deficits, such as amusia, and the co-existed lexical tone difficulties modulate the anatomical structure of the arcuate fasciculus. This is a line of inquiry that will not only inform us of the neural anatomical nature of amusia, but also provide novel insights into the intricate relationship between music and speech pitch processing.

Our work is the first study to reveal a reduced global connectivity efficiency of the brain network in amusics as well as an increase in the global connectivity cost due to co-existing lexical tone deficits. These results provide a holistic perspective on the anatomical substrates underlying amusia in a tone language background.

## Methods

### Participants

Thirty-one amusic individuals (13 males, age range: 17 to 30, mean ± SD: 21.8 ± 3.3) and twenty-four normal controls (10 males, age range: 17 to 25, mean ± SD: 21.9 ± 1.9) matched in gender, age, IQ (based on the Chinese-revised Wechsler Adult Intelligence Scale)^54^, and brain size participated in the current study. All of the participants were native Mandarin speakers and right-handed^55^ and reported no audiological or neurological deficits. None of the participants had any formal music training. Their audiometric thresholds were at or below 20 dB hearing level for octaves ranging from 250 to 8000 Hz. The amusic participants were defined using the Montreal Battery of Evaluation of Amusia^56^, which consists of six subtests, including scale, contour, interval, rhythm, meter, and memory. Each amusic individual scored below the cut-off score of 71.7%, corresponding to two SDs below the mean of the controls according to our previous study^11^. The detailed characteristics of the two groups were summarized in Table 1. In addition, all participants were also screened with the lexical tone perception test, which included tone identification and tone discrimination tasks^11^. The identification subtest includes 192 trails, in which participants need to judge which tone (level/mid-rising/dipping/high-falling) a given word belongs to^11^. The discrimination subtest includes 128 trails, demanding participants to judge whether two tones are the same or different. The average score of the two subtests was taken as an index of the lexical tone perception performance^13^,^14^. Of note, the lexical tone test scores of the amusics were significantly lower than the controls (both *p*s < 0.001).

The current study was approved by the Institutional Review Board at Beijing Normal University and the methods were carried out in accordance with the Declaration of Helsinki. Informed written consent was obtained from each participant.

### Image acquisition

Magnetic Resonance Imaging (MRI) data for all participants were acquired on a Siemens TRIO 3T scanner (Siemans TRIO, Erlangen, Germany). Three-dimensional T1-weighted images with high resolution were obtained by using a three-dimensional rapid acquisition gradient echo sequence that covered the entire brain with the following parameters: 144 sagittal slices, slice thickness = 1.33 mm; repetition time (TR) = 2530 ms; echo time (TE) = 3.39 ms; inversion time = 1100 ms; flip angle = 7°; acquisition matrix = 256 × 256, field of view (FOV) = 256 × 256 mm^2^; and average = 1. Diffusion weighted images were acquired using a single-shot echo planar imaging sequence covering the whole brain with the following parameters: 62 axial slices, slice thickness = 2.2 mm with no interslice gap; TR = 8000 ms; TE = 89 ms; flip angle = 90°; 30 diffusion directions with b = 1000 s/mm^2^; and an additional image without diffusion weighting (i.e., b = 0 s/mm^2^); acquisition matrix = 128 × 128; FOV = 282 × 282 mm^2^; average = 2.

During scanning, participants were instructed to lie down in the supine position with their head snugly fixed by straps and foam pads to minimize head movement.

### Data preprocessing and network construction

Data preprocessing and network construction were performed using PANDA (www.nitrc.org/projects/panda), which is a pipeline toolbox for diffusion MRI analysis^57^. Briefly, the preprocessing procedure included skull-stripping, eddy-current and head-motion correction, fractional anisotropy (FA) calculation, and probabilistic distribution of fibre orientations estimation^52^. Next, we adopted the same procedures used in previous white matter network studies^22^,^23^,^24^ to define network nodes and edges between different nodes.

#### Defining network nodes

Individual T1-weighted images were first co-registered to the FA images in the DTI space using a linear transformation. Next, the T1-weighted image was non-linearly normalized to the ICBM-152 T1 template in the MNI space. Finally, inverse transformations were employed to warp the automated anatomical labelling (AAL) atlas^58^ from the MNI space to the native diffusion space, with preservation of the discrete labelling values using the nearest-neighbour interpolation method. The AAL atlas has been mostly widely used in white matter network research to illustrate the connections between cortical regions^22^,^23^,^59^,^60^. As a result, we obtained 90 cortical and subcortical regions (45 for each hemisphere), each of which represented a network node^15^. Of note, we did not exclude the grey matter voxels in these seed regions, nor did we apply any other transformations.

#### Defining network edges

The edge was defined based on probabilistic tractography^52^, see Supplementary materials for detail. For each defined node, the connectivity probability was computed between it and the remaining 89 nodes. As a result, a 90 × 90 weighted network was generated, the elements of which was the connectivity probability. We selected ten sparsities ranging from 9% to 27% at intervals of 2% according to findings obtained from previous white matter weighted network studies^22^,^23^. The pilot analysis demonstrated that there was no isolated node when the sparsity was equal to or greater than 9%, and the sparsity of all raw matrices was higher than 27%.

#### Unweighted and high-resolution network

To test the effects of weight and resolution, we constructed a binary network and a high-resolution network for each individual. See Supplementary materials for detail.

### Network Properties Analysis

The topological properties of the white matter networks were analysed using graph theory. For each individual, we calculated the network attributes at both the global and regional (nodal) levels.

#### Global-level network analysis

For whole-brain organization, we mainly focused on the clustering coefficient (C_p_), shortest path length (L_p_), and small-world properties (normalized clustering coefficient γ and normalized shortest path length λ)^61^ of the network. The C_p_ is the average of the clustering coefficient of all nodes, which expresses the likelihood that all neighbours of a given node are also interconnected and indicates the local efficiency for the transformation of information^15^. The shortest path length L_p_ is defined as the length of the path between node i and j with the minimal distance, which represents the most efficient information-transfer between the two nodes^15^. Importantly, the length of each edge is not physical but is computed using the weight p_ij_. The shortest path length L_p_ of a network represents the average of the shortest path length over all pairs of nodes. To obtain the normalized clustering coefficient γ and the normalized shortest path length λ, we first generated 1000 random networks matched in the number of nodes, edges, and degree distribution, but preserved the weight distribution of the real network. Next, we computed the average C_random_ and L_random_ over these random networks. Subsequently, the γ (γ = C_p_/C_random_) and λ (λ = L_p_/L_random_) were calculated to represent the normalized clustering coefficient and normalized shortest path length, respectively. A small world network should meet the criteria of γ = C_p_/C_random_ ≫ 1 and λ = L_p_/L_random_ ≈ 1^61^. Given that there was no isolated node in the weighted networks, we did not calculate the local efficiency and global efficiency because they are conceptually similar to the clustering coefficient and shortest path length, respectively^62^.

#### Nodal (regional)*-*level network analysis

To examine the connectivity of the individual node with the remaining nodes in the network, we calculated the strength of each node (nodal strength), which expresses the sum of the weights of all of the neighbouring edges connected to a given node and reflects the importance of the corresponding node in the network^63^. We mainly focus on this property since it is the most direct way to indicate the connective situation between a certain node and all the rest of the nodes, which is similar to the real condition of white matter connection between brain regions. To examine if the pivot nodes of the network were also affected by amusia, we defined hub nodes of both groups. A “hub” means a higher importance of functional integration and a central position of a node in the network^63^. Consistent with previous studies^22^,^60^, we defined the hub according to the nodal strength: a node was considered a hub only if its nodal strength was one standard deviation (SD) above the mean nodal strength of the network. To further explore our data, we also compared the nodal efficiency (representing the ability of transformation of information of a node in the network) and nodal betweenness (representing the number of shortest paths in a network that pass through a given node) between the controls and amusics^63^.

### Statistical Analysis

The group differences of age, brain size, and IQ were tested using parametric tests (independent samples t test for two groups). The ratio of gender was tested using the Pearson chi-square test. The scores of the MBEA and lexical tone test were not normally distributed, and thus, the related group differences were tested using nonparametric (Man-Whitney) tests. To obtain the group difference of global and nodal network properties, the permutation test, which has been widely used in network statistics^22^,^59^, was applied according to the steps described in Supplementary materials. The probability of type I error α = 0.05 (FDR corrected) was used as the significance threshold.

The same between-group comparison procedure of the permutation test was also used for both the binary network and high-resolution network.

To examine the association of white matter network characteristics and behavioural performance of lexical tone perception, we investigated the relationship between the AUC of network properties, which showed significant between-group differences and the lexical tone test score (namely the average score of the tone discrimination subtest and the tone identification subtest) using Spearman correlation.

## Additional Information

**How to cite this article**: Zhao, Y. *et al.* Abnormal topological organization of the white matter network in Mandarin speakers with congenital amusia. *Sci. Rep.* **6**, 26505; doi: 10.1038/srep26505 (2016).

## Supplementary Material

Supplementary Information

## Figures and Tables

**Table 1:** Characteristics of the controls and amusics.

	Controls (*n* = 24)	Amusics (*n* = 31)
Male/Female	10/13	13/18
Age (SD)	21.9 ± 1.9	21.8 ± 3.3
IQ (SD)	125.6 ± 6.0	123.6 ± 6.8
Brain size (SD)	1415.3 ± 110.1	1421.2 ± 106.6
MBEA (SD)	88.019 ± 6.029	64.063 ± 5.185
Tone Test (SD)	97.472 ± 2.891	86.083 ± 14.695

Two groups were matched in gender, age, intelligence quotient (IQ), and brain size (mm^3^). MBEA refers to the Montreal Battery of Evaluation of Amusia. SD indicates standard deviation.
