# Corrigendum: Abnormal topological organization of the white matter network in Mandarin speakers with congenital amusia

**DOI:** 10.1038/srep30102

**Published:** 2016-07-22

**Authors:** Yanxin Zhao, Xizhuo Chen, Suyu Zhong, Zaixu Cui, Gaolang Gong, Qi Dong, Yun Nan

Scientific Reports 6: Article number: 2650510.1038/srep26505; published online: 05232016; updated: 07222016

This Article contains a typographical error in Table 1. In the ‘Controls (n = 24)’ column, the Male/Female value “10/13” should read “10/14”.

