# Supplementary material for: Abnormal topological organization of the white matter network in Mandarin speakers with congenital amusia
Source: Sci Rep. 2016 May 23;6:26505. doi: 10.1038/srep26505 (PMC4876438; doi:10.1038/srep26505)
Supplement: Supplementary Information [file srep26505-s1.doc]

**Abnormal topological organization of the white matter network in Mandarin speakers with congenital amusia**

Yanxin Zhao^#^, Xizhuo Chen^#^, Suyu Zhong, Zaixu Cui, Gaolang Gong, Qi Dong, Yun Nan^*^

State Key Laboratory of Cognitive Neuroscience and Learning & IDG/McGovern Institute for Brain Research

**^*^Correspondence:**

Yun Nan, Ph.D.

State Key Laboratory of Cognitive Neuroscience and Learning,

Beijing Normal University,

19 Xin-Wai St., Hai-Dian District,

Beijing 100875, P.R. China

Phone: +8610-58802742

nany@bnu.edu.cn

^#^These authors contributed equally to this work.

**Supplementary Methods**

**The specific procedure of Defining network edges using probabilistic tractography**

Markov Chain Monte Carlo sampling was first applied to estimate voxel-wise probability density functions (PDFs) with a two-fibre model for each voxel. Next, each defined node was selected as a seed region and 5000 fibres were sampled for every voxel in this region. In total, 5000 × n (n denotes the number of voxels within the seed region) fibres were sampled for a seed region. Finally, for each sampled fibre, the principle diffusion direction was determined from the local PDF and then proceeded 0.5 mm along the principle diffusion direction to a new position. This tracking procedure continued until it reached the cerebral surface or the path loop back to itself. The connectivity probability from the seed region i to the target region j, P_ij_, was calculated as the fibre numbers passing through the target region divided by 5000 × n and was used as the weight of the network .

**The Construction of the binary network and a high-resolution network**

The binary network was basically the same as the above-mentioned weighted network that was expected for weight. The node of the high-resolution network was defined by randomly parcellating the AAL template into 1024 regions with equal sizes. These seed regions only included low FA values. Five sparsities, ranging from 1% to 5% at intervals of 0.01, were chosen according to findings obtained from a previous white matter network study . The pilot analysis revealed that there was no isolated node when the sparsity was equal to or greater than 1%, and the sparsity of all high-resolution raw matrices was higher than 5%.

**The steps of permutation test**

First, the area under curve (AUC), which was used as a cross-thresholds measure for γ, λ, C_p_, L_p_, and nodal strength S was calculated for each participant. Next, the between-group difference of the mean AUC value was computed. Afterwards, each participant’s AUC value was randomly assigned to one group or the other and the difference in mean AUC values between the randomized groups was calculated. This permutation procedure was repeated 10000 times, and the resultant distributions were used to test the null hypothesis that the observed between-group differences were caused by chance.

**Supplementary Figure 1. Group differences under multiple thresholds (sparsities** **ranging from 9% to 27% at intervals of 2%) of λ (A, p < 0.05 under all thresholds), C_p_ (B, p < 0.05 under all thresholds), and nodal strength of IPL (C, p < 0.05, Bonferroni corrected, under 9%, 11%, 13%, 15%, 17%, 21%, 23%)**


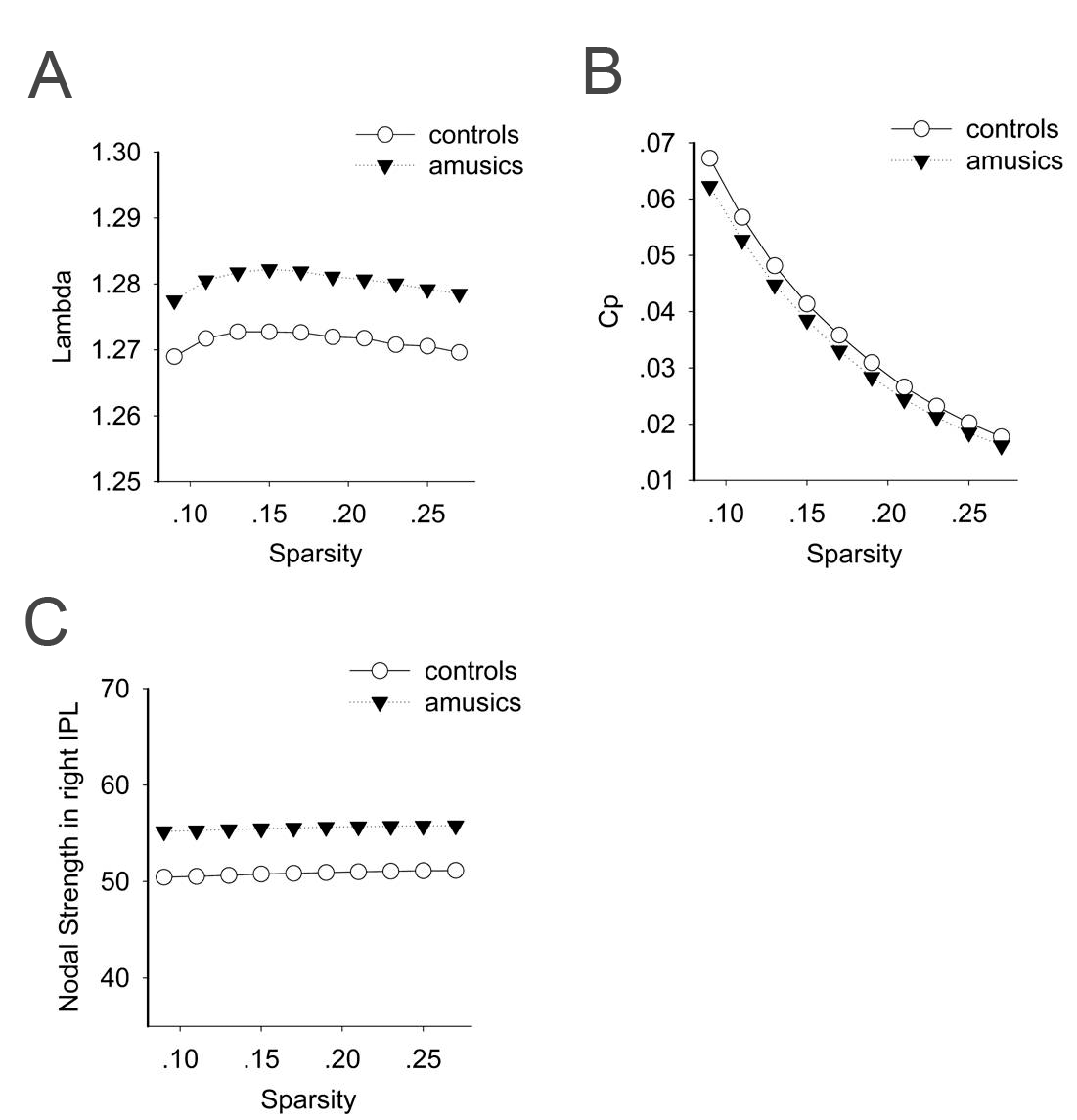


**Reference**

Behrens, T. E., Berg, H. J., Jbabdi, S., Rushworth, M. F., & Woolrich, M. W. (2007) Probabilistic diffusion tractography with multiple fibre orientations: What can we gain? Neuroimage, 34:144-155.

Cao, Q., Shu, N., An, L., Wang, P., Sun, L., Xia, M. R., . . . He, Y. (2013) Probabilistic diffusion tractography and graph theory analysis reveal abnormal white matter structural connectivity networks in drug-naive boys with attention deficit/hyperactivity disorder. J Neurosci, 33:10676-10687.
